# Maternal Gestational Dietary Fat has Minimal Effects on Serum Lipid Profiles and Hepatic Glucose Transporter 2 and No Effect on Glucokinase Expression in Neonatal Wistar Rat Offspring

**Published:** 2011-09

**Authors:** Marlon E. Cerf, Keith Williams, Christo J. Muller, Johan Louw

**Affiliations:** *Diabetes Discovery Platform, South African Medical Research Council, Tygerberg, Cape Town, South Africa*

**Keywords:** fatty acids, fetal programming, insulin, triglycerides

## Abstract

The study investigated the effects of maternal diets, varying in fat content, on lipid profiles and the expression of hepatic glucose transporter 2 (GLUT2) and glucokinase (GK) in neonatal Wistar rat offspring. Dams were maintained on diets of 10% (control), 20% (20F), 30% (30F) and 40% (40F) fat as energy throughout gestation; daily food intakes and weekly body weights were measured. Circulating fasting glucose, insulin and glucagon concentrations were determined in dams and their neonatal offspring. In neonates, total serum triglyceride, total and individual serum fatty acid concentrations, hepatic GLUT2 and GK mRNA and protein expression were determined. In dams, overall food intake of 20F (645.50 ± 25.26 g) and 40F (716.30 ± 14.15 g) dams was reduced compared to control (1007.00 ± 44.83 g) and 30F (924.50 ± 21.16 g) dams. The 20F neonates displayed elevated blood glucose concentrations (4.63 ± 0.153 mmol/l) compared to control neonates (4.14 ± 0.112 mmol/l). In 30F neonates, serum palmitoleic acid was reduced (1.63 ± 0.21% *vs.* 3.56 ± 0.38%) whereas stearic acid was elevated (10.05 ± 0.40% *vs.* 7.40 ± 0.72%) compared to control neonates. Further, the palmitoleic acid/palmitic acid ratio was reduced in 30F neonates (0.085 ± 0.009% *vs.* 0.165 ± 0.020% in control neonates). The 40F neonates displayed elevated GLUT2 immunoreactivity (22.86 ± 0.760%) compared to 20F (11.46 ± 2.701%) and 30F (6.45 ± 1.759%) neonates. Gestational programming with different dietary fat proportions minimally affects lipid profiles and hepatic GLUT2 immunoreactivity in neonatal offspring.

## INTRODUCTION

Inadequate maternal feeding or exposure to deleterious environmental compounds during the critical period of fetal life can alter developmental trajectories (1), structure, physiology and metabolism (2). Maternal diets may have lifelong effects on offspring gene expression and potentially cause susceptibility to complex metabolic diseases (2). Free fatty acids (FFA) are divided into classes: saturated (SFA), monounsaturated (MUFA), polyunsaturated (PUFA), omega 3 (n-3), omega 6 (n-6) and trans fatty acids (TrFA) (3). FFA play a role in both the cellular and molecular mechanisms of insulin resistance, because they are a determinant of the membrane properties that affect insulin sensitivity (4, 5) and act as physiological signaling molecules that induce insulin resistance (6, 7). Circulating FFA increase hepatic glucose output by increasing gluconeogenesis or glycogenolysis (8). Further, elevated FFA concentrations contribute to the pathogenesis of obesity, metabolic syndrome, cardiovascular disease and diabetes. A recent study showed that total plasma FFA were moderately increased and fatty acid composition was markedly altered in male Wistar rats fed a high fat diet over 8 weeks (9).

The liver plays a vital role in the maintenance of blood glucose homeostasis by regulating glucose production. Glucose sensing units are present in different locations of the body and their integrated functions participate in the adaptation of an organism to new metabolic demands (10). Glucose transporter 2 (GLUT2) and glucokinase (GK) represent glucose sensors that are functional in the liver, pancreatic beta cells, kidneys and brain.

We recently demonstrated an increase in brain GLUT2 immunoreactivity in neonates maintained on either a 30% fat or 40% fat diet, with reduced GK immunoreactivity in neonates maintained on a 20% fat diet; no differences in brain GLUT2 or GK mRNA expression were detected in neonates (11). The aim of this study was therefore to investigate, in one-day old neonatal offspring, the effect of maternal diets varying in fat content, on circulating glucose, insulin, glucagon and lipid (triglyceride and FFA) concentrations, and on the expression of hepatic glucose sensing factors by determining GLUT2 and GK mRNA and protein expression, as there has been little research on this important topic. In addition, neonatal gender distribution, liver, pancreas and heart weights were determined. Further, maternal food intake, body weight, organ weights (liver, pancreas, heart and retroperitoneal fat), circulating glucose, insulin and glucagon concentrations were also measured.

## MATERIALS AND METHODS

### Experimental design

Ethical approval was obtained from the institutional (South African Medical Research Council) ethics committee. Three-month old virgin Wistar female rats weighing 220-275 g were individually housed and mated overnight with pregnancy confirmed by the presence of a vaginal plug(s). After confirmation of pregnancy, dams were removed, individually housed and assigned to groups (n=4 per group) maintained on a 10% fat (as energy) diet (control); a 20% fat diet (20F); a 30% fat diet (30F); and a 40% fat diet (40F). The groups therefore comprised control (10% fat diet), 20F, 30F and 40F mothers and their neonatal offspring. Briefly the major macronutrient proportions of the diets were: control (10.69% fat, 15.13% protein, 74.16% carbohydrate); 20F (20.68% fat, 15.09% protein, 64.22% carbohydrate), 30F (31.00% fat, 15.77% protein, 53.23% carbohydrate) and 40F (40.17% fat, 15.09% protein, 44.73% carbohydrate). The diets were in patty form and all dams were maintained on their respective diets throughout gestation.

### Maternal blood sampling and the determination of body weights, food intake and organ weights

The mothers (n=4 per group) were weighed prior to mating; on days 7, 14 and 20 of gestation; and on the day of delivery. Food intake was measured daily. After 3 h of fasting, blood was collected prior to mating and on days 7, 14 and 20 of gestation to measure blood glucose concentrations. On the day of delivery, blood glucose, serum insulin and serum glucagon concentrations were determined.

On the day of delivery, mothers were anesthetized by intraperitoneal injection with 6% sodium pentobarbital (30-40 mg/kg body weight i.e. 0.4-0.5 ml intraperitonealy per 200-250 g rat), the liver, pancreas, heart and retroperitoneal fat were excised followed by exsanguation. The liver, pancreas, heart and retroperitoneal fat weights were weighed on a digital scale measuring up to 10-4 g (n=4 per group). All organ weights were also adjusted for body weight (organ weight/body weight × 100).

### Neonatal gender distribution, blood sampling, liver, pancreas and heart collection

Within 24 h of birth, the gender was identified in the neonatal offspring. Gender distribution was reported as percentages of either males or females per litter with the litter representing 100%.

Blood was collected from individual neonates for the determination of glucose concentrations (n=38-56 per group). For the determination of serum insulin, serum glucagon concentrations, serum triglyceride and serum FFA concentrations, the blood from neonates per litter was pooled (n=4 per group) in order to constitute sufficient volumes for analyses.

Whole liver was rapidly excised, weighed (n=38-56 per group) and then either snap-frozen at -800C for quantification of mRNA expression (n=10 per group for quantitative real-time (RT)-PCR) or fixed in buffered formalin for the determination of protein expression (n=4 per group for immunohistochemistry and image analysis) of GLUT2 and GK. Neonatal pancreata and hearts (n=38-53 per group) were also excised and weighed. All organ weights were also adjusted for body weight (organ weight/body weight X 100).

### Determination of blood glucose, serum insulin, glucagon, triglyceride and FFA concentrations

In mothers and neonates, blood glucose concentrations were determined with a glucometer (Precision QD, Medisense Inc, Oxfordshire, UK) employing the oxidase method. Radio-immunoassay techniques were applied to determine serum insulin (^125^I-labelled rat anti-insulin, Linco, St. Charles, Missouri) and serum glucagon (^125^I-labelled anti-glucagon, Linco, St. Charles, Missouri) concentrations. I-labeled samples were counted in a Perkin Elmar 1470 automatic gamma counter (Perkin Elmar, Turku, Finland).

In neonates, serum triglyceride concentrations were determined by the GPO-PAP method using an enzymatic colorimetric test with lipid clearing factor and measured in an autohumalyzer A5 (Human Diagnostics, Wiesbaden, Germany). In addition, total and individual serum FFA concentrations were determined by rapid thin-layer chromatographic-gas-liquid (12). A total of twenty individual FFA were measured. Each individual FFA represented a percentage of the total FFA concentrations. Further, the ratios of palmitoleic acid (16:1 n-7)/palmitic acid (16:0) and oleic acid (18:1 n-9)/stearic acid (18:0) were determined as alterations in these ratios suggest insulin resistance.

### Quantitative RT-PCR

Whole liver was harvested and snap frozen at -80°C in liquid nitrogen for molecular biology studies. Hepatic tissue was homogenized for RNA extraction with Tri-reagent (Sigma, St. Louis, Missouri). RNA was purified using an RNeasy mini kit as recommended by the manufacturers (Qiagen, Valencia, California) and was cleared from DNA using DNAse treatment. RNA integrity was assessed with a Nanodrop ND-1000 spectrophotometer (Nanodrop Technologies, Wilmington, Delaware), an Agilent Bioanalyser and the Agilent RNA 6000 Nano kit (Agilent, St. Clara, California).

cDNA synthesis was performed using a high capacity cDNA kit (Applied Biosystems, Foster City, California). RNA samples were prepared by pipeting 2 μg of RNA per sample and constituting the sample to 10 μl with water. A positive control was prepared by constituting 2 μg of RNA control template to 10 μl with water; and a negative control by adding 10 μl of water to a PCR tube. All PCR tubes were prepared in duplicate and placed in a thermal cycler (Applied Biosystems 2720, Foster City, California).

Primers were designed using Primer Express (Applied Biosystems, Foster City, California) and the gene database at www.ncbi.nlm.nih.gov. The primer sequences were:

GLUT2 forward: 5’-CTGTCTGTGTCCAGCTTTGCA-3’
GLUT2 reverse: 5’-CAAGCCACCCACCAAAGAAC-3’
GK forward: 5’-CAGTGGAGCGTGAAGACAAA-3’
GK reverse: 5’-AGGGAAGGAGAAGGTGAAGC-3’

Quantitative RT-PCR was performed using the Applied Biosystems 7500 RT-PCR system and the Power SYBR Green PCR kit (Applied Biosystems, Foster City, California) using a cycler program consisting of an activation step of 10 min at 95°C, 40 cycles with a 15 s denaturing step at 95°C and 1 min at 60°C for annealing and extension. Both GAPDH and ActB were used as house-keeping genes. Gene expression was normalized against both house-keeping genes and expressed as actual expression divided by the average of the expression of the house-keeping genes. Data analysis was performed with 7500 SDS software to run a Relative Quantification plate and to analyze the RT-PCR results.

### Immunohistochemistry

Harvested livers were fixed in 4% buffered formaldehyde for 12 h, processed, embedded in paraffin wax, sectioned and sections were mounted onto aminosaline (APES) coated slides. For immunohistochemical studies and image analysis (n=4 per group), liver sections were immunostained for GLUT2 (1:100; WAK-Chemie, Bad Soden, Germany) and GK (1:100; a kind gift from Prof Shiota, Vanderbilt University, Tennessee). All sections were incubated with ABC complex (Vector Laboratories, Burlingame, California) for 60 min at room temperature, washed in 50 mM Tris buffer for 10 min and stained with 0.05% diaminobenzadine containing 0.01% H_2_O_2_ for 5 min at room temperature. Sections were counterstained with Mayer’s hematoxylin for 2 min, left to dry and mounted in entellan. The primary antibody was omitted for method controls.

### Semi-quantitative image analysis

Image analysis was performed with Leica Qwin Pro version 3.0 image analysis software (Leica, Wetzlar, Germany) attached to a Leica DC290 digital camera (Leica, Wetzlar, Germany) mounted onto an Olympus BX60 light microscope (Olympus, Hamburg, Germany). A macro was specifically developed to capture the images for the detection and quantification of immunohistochemicaly positive areas. The macro allowed visual assessment of the areas detected that could be adjusted interactively to compensate for slide to slide variation of staining. All images were captured using a ×20 objective and stored on a personal computer at a final resolution of 1024 × 796. GLUT2 and GK immunoreactivity were quantified using RGB color thresholding. The area of GLUT2 and GK immunoreactivity was expressed as a percentage of the total hepatic tissue per field.

### Statistical analysis

Comparisons between the groups were analyzed using One-way ANOVA followed by Bonnferroni’s post-test. Significance was established at *p*<0.05. Data are reported as means ± SEM.

## RESULTS

### Maternal food intake, body weight and litter size

The 20F mothers had reduced food consumption during the first, second and third weeks of gestation compared to both control and 30F mothers (Table 1). The 40F mothers consumed less food during the first week of gestation compared to control mothers; and during the second and third weeks of gestation compared to control and 30F mothers (Table 1). Further, overall food intake of 20F and 40F mothers was reduced compared to both control and 30F mothers (Table 1). There were no differences in maternal body weights throughout gestation, except on day 20 of gestation when 30F mothers were heavier compared to 20F mothers (Table 1). There were no significant differences in litter size (Table 1) although 20F mothers produced relatively fewer offspring.

**Table 1:** Maternal food intake and body weight during gestation and litter size

	Control	20F	30F	40F

Food intake (g)				
Week 1 of gestation	299.80 ± 15.90	211.30 ± 8.96^a^^c^	268.30 ± 6.09	233.80 ± 9.44^a^
Week 2 of gestation	333.30 ± 19.44	200.80 ± 6.63^a^^c^	306.30 ± 7.30	237.80 ± 17.64^a^^c^
Week 3 of gestation	384.30 ± 16.36	233.50 ± 11.72^a^^c^	350.00 ± 7.94	244.80 ± 14.16^a^^c^
Overall food intake	1007.00 ± 44.83	645.50 ± 25.26^a^^c^	924.50 ± 21.16	716.30 ± 14.15^a^^c^
Body weight (g)				
Before pregnancy	246.80 ± 5.52	238.00 ± 12.79	268.25 ± 5.14	243.50 ± 11.59
Day 7 of gestation	266.80 ± 9.96	260.50 ± 14.17	295.58 ± 7.64	272.30 ± 7.87
Day 14 of gestation	301.00 ± 13.23	286.50 ± 13.11	332.30 ± 7.83	303.00 ± 9.77
Day 20 of gestation	375.00 ± 16.14	350.00 ± 13.79	415.50 ± 7.29^b^	370.70 ± 8.74
Day of delivery	286.80 ± 8.41	253.70 ± 1.33	290.00 ± 12.42	315.00 ± 26.74
Litter size	13.25 ± 1.18	9.75 ± 2.06	13.25 ± 0.48	13.00 ± 1.08

Data are means ± SEM. (n=4). 20F, 20% fat diet; 30F, 30% fat diet; 40F, 40% fat diet.

a *p*<0.05 *vs*. control;

b *p*<0.05 *vs*. 20F;

c *p*<0.05 *vs*. 30F.

### Maternal organ weights

No significant differences were found in either unadjusted or adjusted maternal liver, pancreas, heart or retroperitoneal fat weights (Table 2).

**Table 2:** Maternal liver, pancreas, heart and retroperitoneal fat weight (unadjusted and adjusted for body weight)

	Control	20F	30F	40F

Liver weight (g)	9.71 ± 0.38	9.53 ± 0.25	11.12 ± 0.31	10.45 ± 0.52
Adjusted liver weights	3.389 ± 0.114	3.745 ± 0.155	3.531 ± 0.0545	3.347 ± 0.126
Pancreas weight (g)	0.92 ± 0.19	1.13 ± 0.15	0.94 ± 0.16	1.14 ± 0.41
Adjusted pancreas weights	0.328 ± 0.061	0.417 ± 0.075	0.304 ± 0.063	0.366 ± 0.138
Heart weight (g)	0.91 ± 0.09	0.94 ± 0.07	0.99 ± 0.02	0.84 ± 0.04
Adjusted heart weights	0.317 ± 0.025	0.346 ± 0.012	0.315 ± 0.013	0.271 ± 0.014
Retroperitoneal fat weight (g)	6.20 ± 2.51	5.55 ± 0.97	6.94 ± 1.89	8.21 ± 0.67
Adjusted retroperitoneal fat weights	2.167 ± 0.882	2.473 ± 0.380	2.295 ± 0.675	2.668 ± 0.324

Data are means ± SEM. (n=4). 20F, 20% fat diet; 30F, 30% fat diet; 40F, 40% fat diet.

### Maternal blood glucose, serum insulin and serum glucagon concentrations

**Figure 1:**
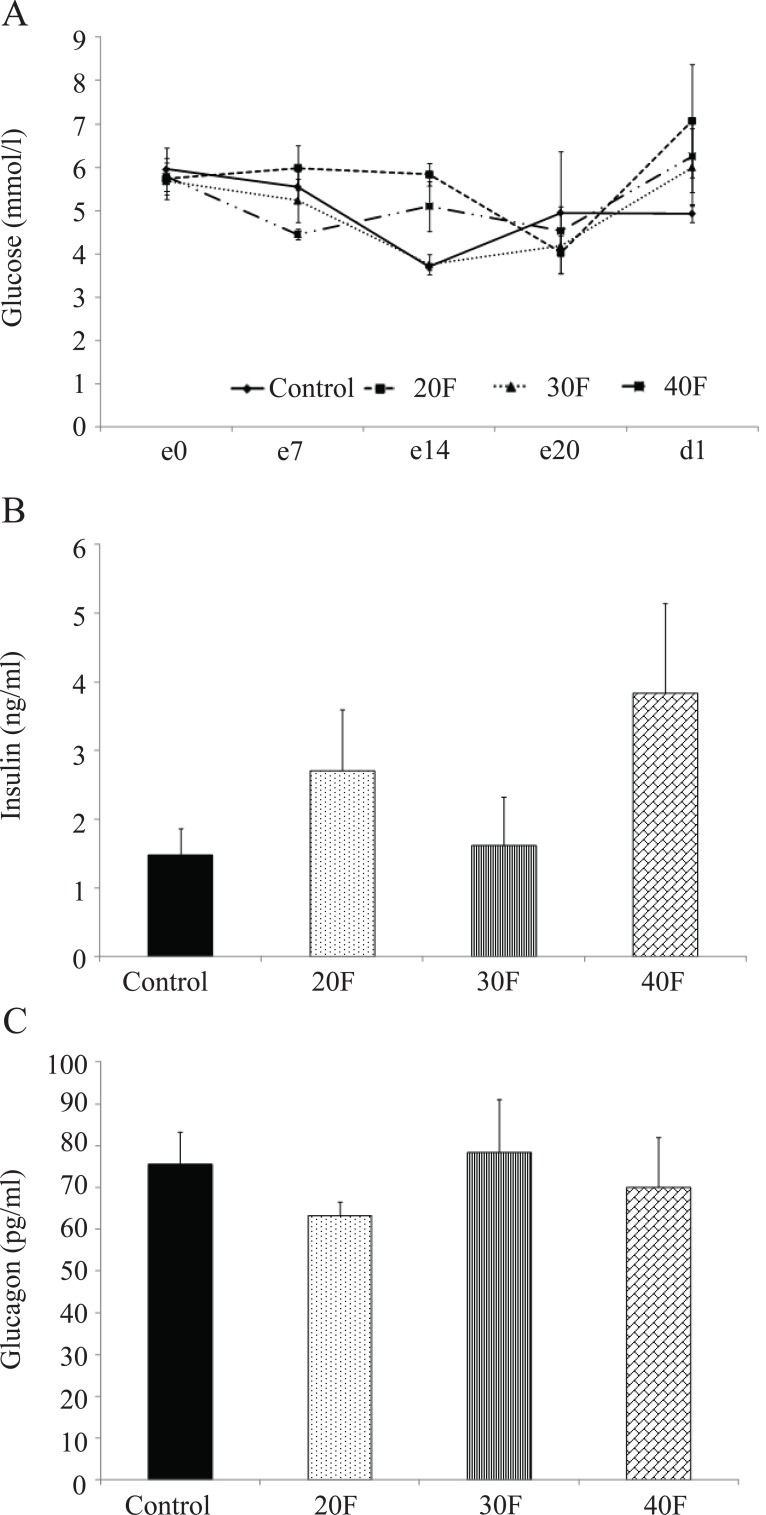
Maternal blood glucose, serum insulin and serum glucagon concentrations. (A), Maternal blood glucose concentrations during pregnancy; (B), Maternal serum insulin concentrations on the day of delivery; (C), Maternal serum glucagon concentrations on the day of delivery. Data are means ± SEM. (n=4). 20F, 20% fat diet; 30F, 30% fat diet; 40F, 40% fat diet.

There were no differences in maternal blood glucose concentrations before gestation, during gestation or on the day of delivery (Fig. 1A). Further, on the day of delivery, serum insulin (Fig. 1B) and serum glucagon (Fig. 1C) concentrations were not significantly altered.

### Neonatal gender distribution and organ weights

There were no significant differences in gender distribution amongst the groups (Table 3). The 20F and 30F neonates were heavier than both control and 40F neonates with no differences detected in the body weights of 40F neonates compared to control neonates (11).

**Table 3:** Neonatal gender distribution, liver, pancreas, heart and brain weights in neonatal rats (unadjusted and adjusted for body weight)

	Control	20F	30F	40F

Male (%)	46.28 ± 4.91	34.24 ± 13.14	54.67 ± 2.84	50.88 ± 8.58
Female (%)	53.72 ± 4.91	65.76 ± 13.14	45.33 ± 2.84	49.12 ± 8.58
Liver weights (g)	0.200 ± 0.005	0.215 ± 0.008	0.220 ± 0.008	0.225 ± 0.007
Adjusted liver weights	3.342 ± 0.077	3.223 ± 0.085	3.180 ± 0.091	3.671 ± 0.111^b^^c^
Pancreas weights (g)	0.0128 ± 0.0004	0.0147 ± 0.0007	0.0156 ± 0.0013	0.0139 ± 0.0005
Adjusted pancreas weights	0.214 ± 0.007	0.225 ± 0.013	0.232 ± 0.024	0.228 ± 0.008
Heart weights (g)	0.0343 ± 0.0007	0.0353 ± 0.0010	0.0355 ± 0.0011	0.0303 ± 0.0009^a^^b^^c^
Adjusted heart weights	0.569 ± 0.009	0.532 ± 0.013	0.515 ± 0.013^a^	0.492 ± 0.013^a^
Brain weights (g)	0.217 ± 0.006	0.196 ± 0.007	0.225 ± 0.007^b^^d^	0.199 ± 0.005 (11)
Adjusted brain weights	3.557 ± 0.113	2.912 ± 0.146^a^	3.175 ± 0.131	3.258 ± 0.092

Data are means ± SEM. (n=53) for control, 38 for 20F, 53 for 30F and 52 for 40F. 20F, 20% fat diet; 30F, 30% fat diet; 40F, 40% fat diet.

a *p*<0.05 *vs.* control;

b *p*<0.05 *vs.* 20F;

c *p*<0.05 *vs.* 30F;

d *p*<0.05 *vs.* 40F.

There were no differences in neonatal liver and pancreas weights (Table 3). When adjusted for body weight, 40F neonates had increased liver weights compared to the 20F and 30F neonates with no further differences amongst the groups. Heart weights were significantly reduced in 40F neonates compared to control, 20F and 30F neonates (Table 3). When adjusted for body weight, both 30F and 40F neonates had reduced heart weights compared to the control neonates. Brain weights of 30F neonates were heavier compared to 20F and 40F neonates with no other significant differences (11). When adjusted for body weight, only the brain weights of the 20F neonates were reduced compared to control neonates (Table 3).

### Neonatal blood glucose, serum insulin, glucagon, triglyceride and FFA concentrations

**Figure 2:**
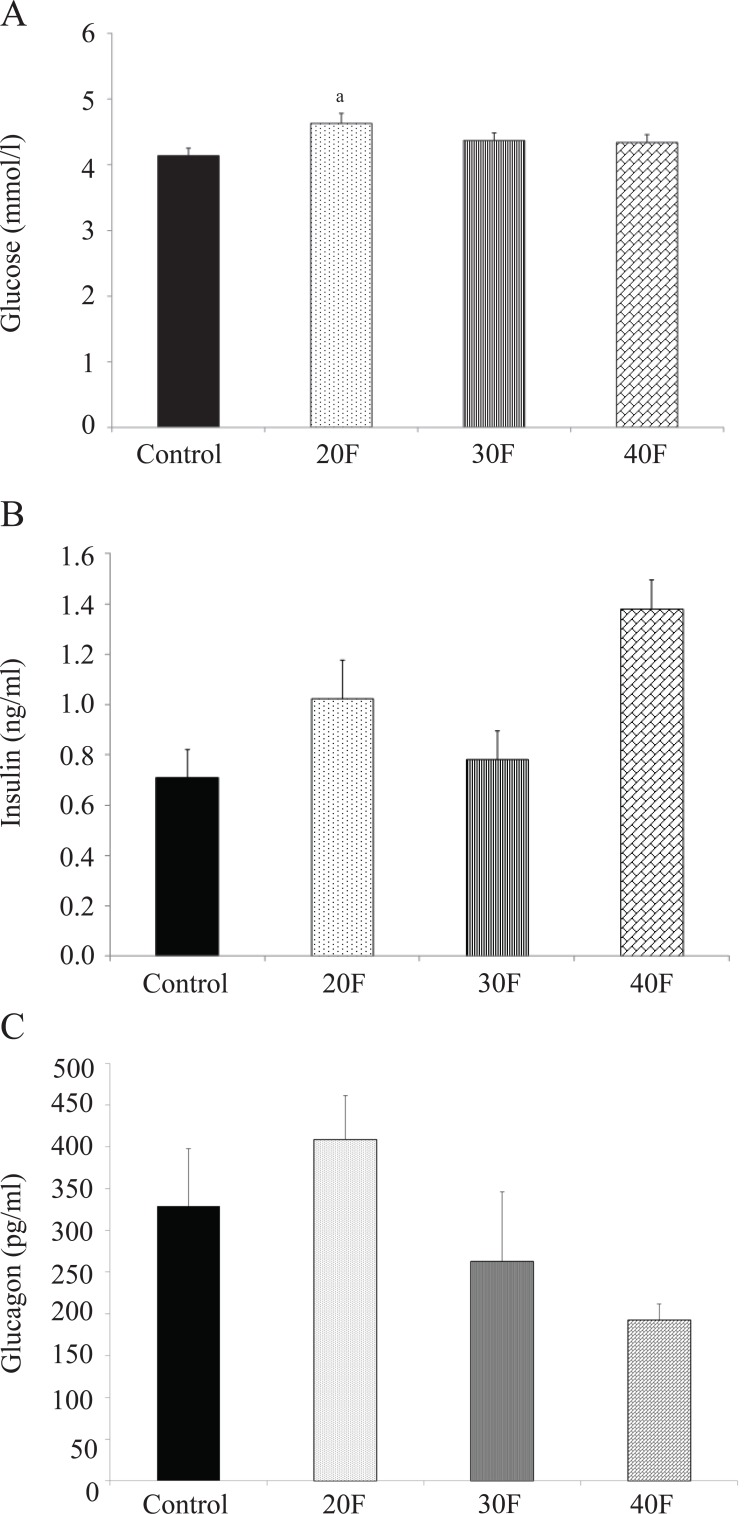
Neonatal blood glucose, serum insulin and serum glucagon concentrations. (A), Neonatal blood glucose concentrations; (B), Neonatal serum insulin concentrations; (C), Neonatal serum glucagon concentrations. Data are means ± SEM. n=53 for control, 38 for 20F, 53 for 30F and 52 for 40F for glucose concentrations; n=4 for insulin and glucagon concentrations. 20F, 20% fat diet; 30F, 30% fat diet; 40F, 40% fat diet. ^a^*p*<0.05 vs. control.

The 20F neonates displayed elevated blood glucose concentrations compared to control neonates (Fig. 2A). There were no differences in the serum insulin (Fig. 2B) and serum glucagon concentrations (Fig. 2C).

The total triglyceride and total FFA concentrations were not significantly altered after dietary intervention (Table 4). However, palmitoleic acid was reduced whereas stearic acid was elevated in 30F neonates compared to control neonates (Table 4). Further, the palmitoleic acid/palmitic acid ratio was reduced in 30F neonates compared to control neonates (Table 4). The ratio of oleic acid/stearic acid remained unchanged (Table 4).

**Table 4:** Total serum triglyceride concentrations, total free fatty acid concentrations and individual free fatty acid percentages in neonatal offspring

	Control	20F	30F	40F

Triglycerides (mmol/l)	1.65 ± 0.26	2.85 ± 0.88	2.26 ± 0.64	1.69 ± 0.20
Total FFA (μg/ml)	2134 ± 253.9	2553 ± 245.1	2632 ± 384.9	2301 ± 509.9
Individual FFA				
14:0 (%)	2.05 ± 0.37	1.80 ± 0.21	1.55 ± 0.11	1.91 ± 0.26
16:0 (%)	21.95 ± 1.16	21.72 ± 0.15	19.65 ± 0.80	21.86 ± 0.72
16:1 n-7 (%)	3.56 ± 0.38	2.48 ± 0.39	1.63 ± 0.21^a^	2.84 ± 0.18
18:0 (%)	7.40 ± 0.72	8.72 ± 0.65	10.05 ± 0.40^a^	8.16 ± 0.54
18:1 n-9 (%)	28.75 ± 2.96	22.98 ± 0.73	23.80 ± 1.42	24.56 ± 1.80
18:2 n-6 (%)	13.30 ± 0.33	13.66 ± 1.06	13.91 ± 1.52	14.83 ± 1.07
18:3 n-3 (%)	1.17 ± 0.02	1.09 ± 0.12	1.07 ± 0.09	1.20 ± 0.09
18:3 n-6 (%)	0.61 ± 0.08	0.63 ± 0.08	0.52 ± 0.06	0.51 ± 0.06
20:0 (%)	0.23 ± 0.09	0.15 ± 0.09	0.28 ± 0.07	0.26 ± 0.09
20:1 n-9 (%)	0.62 ± 0.05	0.52 ± 0.05	0.52 ± 0.05	0.57 ± 0.03
20:2 n-6 (%)	0.51 ± 0.03	0.56 ± 0.05	0.50 ± 0.05	0.56 ± 0.03
20:3 n-6 (%)	0.98 ± 0.02	1.05 ± 0.04	1.11 ± 0.06	0.94 ± 0.03
20:4 n-6 (%)	9.88 ± 0.76	13.10 ± 0.44	13.89 ± 1.28	11.43 ± 1.50
20:5 n-3 (%)	0.73 ± 0.08	0.92 ± 0.08	0.74 ± 0.06	0.63 ± 0.08
22:4 n-6 (%)	2.18 ± 0.17	2.72 ± 0.13	2.22 ± 0.27	2.53 ± 0.46
22:5 n-6 (%)	0.58 ± 0.07	0.74 ± 0.12	1.17 ± 0.29	0.63 ± 0.09
24:0 (%)	0.10 ± 0.05	0.09 ± 0.03	0.20 ± 0.02	0.12 ± 0.04
24:1 n-9 (%)	0.09 ± 0.03	0.11 ± 0.04	0.15 ± 0.02	0.11 ± 0.07
22:5 n-3 (%)	1.47 ± 0.25	1.97 ± 0.28	1.57 ± 0.22	1.60 ± 0.31
22:6 n-3 (%)	3.85 ± 0.50	5.01 ± 0.22	5.45 ± 0.57	4.79 ± 1.06
Ratios				
16:1 n-7:16:0	0.165 ± 0.020	0.112 ± 0.018	0.085 ± 0.009^a^	0.130 ± 0.008
18:1 n-9:18:0	4.033 ± 0.679	2.660 ± 0.117	2.395 ± 0.233	3.085 ± 0.438

Data are means ± SEM. (n=4) for lipid analysis. 20F, 20% fat diet; 30F, 30% fat diet; 40F, 40% fat diet.

a *p*<0.05 *vs.* control.

### Neonatal hepatic GLUT2 and GK mRNA expression and immunoreactivity

**Figure 3:**
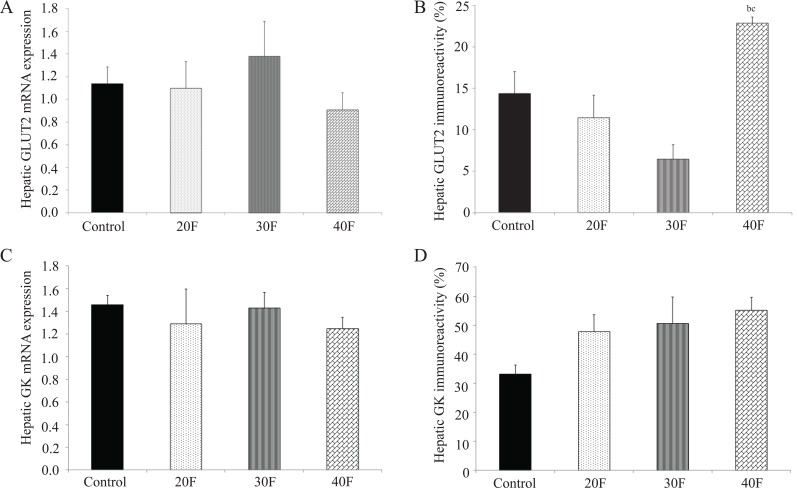
Neonatal hepatic GLUT2 and GK mRNA expression and immunoreactivity. (A), Neonatal hepatic GLUT2 mRNA expression; (B), Neonatal hepatic GLUT2 immunoreactivity; (C), Neonatal hepatic GK mRNA expression; (D), Neonatal hepatic GK immunoreactivity. Data are means ± SEM. n=10 for mRNA expression; n=4 for immunoreactivity. 20F, 20% fat diet; 30F, 30% fat diet; 40F, 40% fat diet. ^b^*p*<0.05 vs. 20F; ^c^*p*<0.05 vs. 30F.

Neonatal hepatic GLUT2 mRNA expression was not altered after dietary fat exposure (Fig. 3A). GLUT2 immunoreactivity did not differ in the experimental neonates (20F, 30F and 40F) when compared to control neonates (Fig. 3B). However, 40F neonates displayed elevated GLUT2 immunoreactivity compared to 20F and 30F neonates (Fig. 3B). There were no differences in either GK mRNA expression (Fig. 3C) or GK immunoreactivity (Fig. 3D) between the groups.

## DISCUSSION

Early disruption of the fatty acid balance in the plasma or liver may influence subsequent abnormalities in glucose metabolism that are associated with the pathogenesis of obesity (13). In the present study, modest effects on the offspring lipid profile were demonstrated. Gestational programming with diets varying in fat (as energy) had no effect on neonatal offspring gender distribution, total triglyceride and total FFA concentrations. However, when analyzing FFA at an individual level some changes presented. Plasma phospholipid, dietary palmitoleic acid and the ratio of palmitoleic acid/palmitic acid were positively associated with diabetes risk (14). Further, circulating palmitoleic acid concentrations correlate positively with insulin sensitivity, with high palmitoleic acid concentrations predicting greater insulin sensitivity (15). In 30F neonates, both circulating palmitoleic acid content and the palmitoleic acid/palmitic acid ratio were reduced potentially reflecting insulin insensitivity. Stearic acid is a major constituent of red meat and confectionary products and contributes 3.5-4% total energy, 10% of the dietary energy derived from fat and 25% of energy derived from saturated fatty acids; palmitic acid accounts for >60% (16). The 30F neonates grew rapidly displaying increased crown to rump length, head dimensions (head length and head width) and body weight (11) suggesting that elevated circulating stearic acid may be associated with enhanced fetal growth.

The elevated glucose concentrations in 20F neonates did not reflect overt hyperglycemia as the glucose concentrations were within the normal range. Hepatic GLUT2 mRNA expression is dependent on nutritional status (17). Pancreatic GK immunoreactivity was reduced in 40F neonates (18) with elevated brain GLUT2 immunoreactivity in both 30F and 40F neonates (11). However, in the present study, no differences were found in hepatic GLUT2 or GK mRNA expression or immunoreactivity, apart from elevated immunoreactivity for GLUT2 in 40F neonates compared to 20F and 30F neonates. Perhaps adaptive mechanisms in the pancreas and brain are in effect, while, in the liver, the expression other isoforms of GLUT and GK may instead be altered.

Altered organ weights (liver, pancreas, heart and brain) presented after adjustment for body weight. Particularly during fetal programming, growth and developmental trajectories are affected which influences organ dynamics: both structurally and physiologically. Therefore it is important to adjust organ weights to body weight to determine organ weight changes more accurately particularly in neonates. The greater degrees of fetal overgrowth and increased body weight-adjusted fetal organ weights (heart, pancreas and liver) observed in obese overfed sheep fetuses suggested a greater amount of excess energy substrate was redistributed to feto-placental tissues in these fetuses, fuelling fetal growth instead of continued maternal fat deposition in animals already with high fat stores (19). This may overburden fetal metabolism with glucose in excess of their developmental requirements and beyond what can be stored as fat, causing altered organ development (19). The cardiac hypotrophy in 40F neonates reflects programming effects and is indicative of stunting, potentially a result of organ sparing. Intrauterine growth restriction by maternal protein restriction resulted in a reduction in the number of cardiomyocytes per heart in neonatal rat offspring (20). In 40F neonates, the cardiac hypotrophy may be associated with the structural and functional changes that characterize programming.

The litter sizes were similar, apart from 20F mothers producing fewer pups (although non-significantly) which may partly explain the reduced food intake of 20F mothers. Diet selection is influenced by macronutrient content, texture and palatability (21). Palatability-driven food intake is linked to obesity (22) and suggested to drive hyperphagia (23). For 40F mothers, the reduced food intake may suggest reduced palatability of the 40F diet relative to the control and 30F diets or that less of the diet is ingested to achieve satiety.

In conclusion, the majority of FFA analyzed (90%) were not altered by nutritional programming reflecting a minor effect of gestational programming by dietary fat intervention. Further FFA analysis and investigation of factors regulating hepatic physiology in the target tissues of insulin may reveal information to further our understanding of metabolic diseases.
